# VASP Activation via the Gα13/RhoA/PKA Pathway Mediates Cucurbitacin-B-Induced Actin Aggregation and Cofilin-Actin Rod Formation

**DOI:** 10.1371/journal.pone.0093547

**Published:** 2014-04-01

**Authors:** Yan-Ting Zhang, Li-Hui Xu, Qun Lu, Kun-Peng Liu, Pei-Yan Liu, Fang Ji, Xiao-Ming Liu, Dong-Yun Ouyang, Xian-Hui He

**Affiliations:** 1 Department of Immunobiology, Jinan University, Guangzhou, China; 2 Department of Cell Biology, Jinan University, Guangzhou, China; 3 Department of Anatomy and Cell Biology, East Carolina University Brody School of Medicine, Greenville, North Carolina, United States of America; 4 Department of Obstetrics and Gynecology, The First Affiliated Hospital of Jinan University, Guangzhou, China; 5 Guangdong Entomological Institute, Guangzhou, China; 6 Southern China Primate Research Center, Guangzhou, China; Institut national de la santé et de la recherche médicale - Institut Cochin, France

## Abstract

Cucurbitacin B (CuB), a potent antineoplastic agent of cucurbitacin triterpenoids, induces rapid disruption of actin cytoskeleton and aberrant cell cycle inhibiting carcinogenesis. However, the underlying molecular mechanism of such anticancer effects remains incompletely understood. In this study, we showed that CuB treatment rapidly induced vasodilator-stimulated phosphoprotein (VASP) phosphorylation (i.e. activation) at the Ser157 residue and generated VASP clumps which were co-localized with amorphous actin aggregates prior to the formation of highly-ordered cofilin-actin rods in melanoma cells. Knockdown of VASP or inhibition of VASP activation using PKA-specific inhibitor H89 suppressed CuB-induced VASP activation, actin aggregation and cofilin-actin rod formation. The VASP activation was mediated by cAMP-independent PKA activation as CuB decreased the levels of cAMP while MDL12330A, an inhibitor of adenylyl cyclase, had weak effect on VASP activation. Knockdown of either Gα13 or RhoA not only suppressed VASP activation, but also ameliorated CuB-induced actin aggregation and abrogated cofilin-actin rod formation. Collectively, our studies highlighted that the CuB-induced actin aggregation and cofilin-actin rod formation was mediated via the Gα13/RhoA/PKA/VASP pathway.

## Introduction

Cucurbitacins are triterpenoid compounds isolated from Cucurbitaceae plants that have been used as folk medicines for centuries. They possess a broad spectrum of pharmacological properties including cancer chemoprevention and anticancer activity [1]–[4]. Several anticancer mechanisms of cucurbitacins have been identified, such as inhibition of Janus kinase/signal transducer and activator of transcription-3 (JAK/STAT3) signaling [5]–[8], interference with mitogen-activated protein kinases (MAPKs) pathway [6], [8], [9], induction of apoptosis [6], [7], [10], [11], and disruption of the actin cytoskeleton [9]–[17]. However, the precise action target(s) for cucurbitacins is currently unknown. Although cucurbitacins, including cucurbitacin B (CuB), are known as insect steroid hormone antagonists acting on the ecdysteroid receptor [18], the possibility that cucurbitacins target the ecdysteroid receptor in mammalian cells could be excluded due to the lack of such receptors in those cells [4]. Recently, increasing evidence indicates that the actin cytoskeleton may be the common upstream target for cucurbitacins to exert their pharmacological properties, leading to cell deformation, multinucleated cell formation, cell cycle arrest and cell motility inhibition [10], [12], [13].

Although the effect of cucurbitacins on the actin cytoskeleton has been observed two decades ago [19] and studies repeatedly demonstrate a rapid disruption of actin cytoskeleton by cucurbitacins, the precise mechanism is still incompletely understood. Several recent reports have shed new light on their action mechanism for such actions. For example, some researchers suggest that cucurbitacin E directly interacts with actin and consequently stabilize the polymerized actin, although such an effect was weak [20]. Others suggest that cucurbitacin causes actin aggregation by indirectly interfering with actin dynamics [13]. In supporting this, another study indicated that cofilin, a critical actin-depolymerizing factor, might be a target of cucurbitacins because it could bind to a synthesized biotin-linked cucurbitacin E, and could be activated (i.e. dephosphorylated) by cucurbitacin E and I [21]. This observation has been corroborated by our recent studies showing that both CuB and cucurbitacin IIb induced cofilin activation in melanoma cells [17], Jurkat cells [14] and prostate cancer cells [22]. Interestingly, cofilin-actin rods were formed after exposure to cucurbitacin IIb for more than 4 h, while cell deformation and actin aggregation were induced much earlier [22]. Consistent with its action on the actin cytoskeleton, CuB substantially changed the expression of many actin-regulating factors as revealed by quantitative proteomic analysis [12]. It is worth noting that CuB treatment could upregulate the expression of vasodilator-stimulated phosphoprotein (VASP) [12], suggesting that it has a potential role in CuB-induced disruption of the actin cytoskeleton.

VASP is an important member of Enabled/vasodilator-stimulated phosphoprotein (Ena/VASP) family [23]. Several multi-functional actin-modulating proteins belong to this family, which localize at regions where actin is dynamically remodeled and new filaments are formed [24], [25]. VASP constitutively or stimulus-dependently localizes at the filopodia or lamellipodia, acting as an F-actin barbed end binding protein to facilitate actin filament elongation by interacting with G-actin, profilin and other actin regulatory factors [26], [27]. VASP can also directly regulate the assembly of the actin-filament network, modulates the morphology and behavior of membrane protrusion structures, and functions in cell migration and adhesion [23], [24], [28]. These observations support that VASP plays a critical role in remodeling actin cytoskeleton in cells. As VASP has an essential role in linking signaling pathways to remodeling the actin cytoskeleton, we hypothesized that VASP might participate in the process of actin aggregation and reorganization induced by cucurbitacins. In this study, we sought to explore the role of VASP in CuB-induced disruption of actin cytoskeleton in human A375 and mouse B16F10 melanoma cell lines. Our results provided evidence that CuB induces actin aggregation and cofilin-actin rod formation through activation of the Gα13/RhoA/PKA/VASP pathway.

## Materials and Methods

### Chemicals

Cucurbitacin B (molecular weight 558.7 Da) with 98% purity was obtained from Zhongxin Innova Laboratories (Tianjin, China), dissolved in dimethyl sulfoxide (DMSO) at 10 mM, and stored at −20°C. N-acetyl-L-cysteine (NAC), H89, MDL12330A, dithiothreitol (DTT), propidium iodide, and DMSO were from Sigma-Aldrich (St Louis, MO, USA). RNase A was purchased from Invitrogen (Carlsbad, CA, USA). Forskolin was obtained from Calbiochem/Millipore (Billerica, MA, USA).

### Cell culture

Human A375 and murine B16F10 melanoma cell lines were obtained from the Cell Bank of the Chinese Academy of Sciences (Shanghai, China). Cells were cultured in DMEM (Invitrogen) supplemented with 10% fetal bovine serum (FBS) (Invitrogen), 100 U/ml penicillin, and 100 μg/ml streptomycin (Invitrogen), and maintained at 37°C in a humidified incubator of 5% CO_2_.

### Immunofluorescence microscopy

After incubation with CuB (0.1 μM), cells were fixed in 4% paraformaldehyde prepared in phosphate-buffered saline (PBS), permeabilized with ice-cold 100% methanol, and immunostained with mouse anti-β-actin (1∶500; Cell Signaling Technology) or rabbit anti-VASP (1∶300; Cell Signaling Technology), followed by CF488-conjugated goat-anti-mouse IgG (1∶700) or CF568-conjugated goat-anti-rabbit IgG (1∶700), highly cross-absorbed (Biotium, Hayward, CA, USA). Nuclei were revealed by Hoechst33342 (5 μg/ml) staining. Fluorescence images were observed and collected under a Leica DMIRB fluorescent microscope (Leica Microsystems, Wetzlar, Germany) armed with a Spinning Disk Confocal Microscopy system (UltraView cooled CCD; Perkin Elmer, Waltham, MA, USA).

### Co-localization analysis

All fluorescence image analysis was conducted using ImageJ software (National Institutes of Health, Bethesda, MD). Co-localization analysis was performed using intensity correlation analysis (ICA) method as described previously [29]. The PDM value is the Product of the Differences from the Mean, i.e. for each pixel: PDM  =  (red intensity – mean red intensity × (green intensity – mean green intensity). PDM images were created using a plugin for ImageJ found at http://www.uhnresearch.ca/facilities/wcif/imagej/colour_analysis.htm#coloc_coeff. ICA produces a PDM image of graded co-localization, where positive PDM value correspond to a high degree of co-localization (here maximum values shown in yellow, negative values shown in blue). The Intensity Correlation Quotient (ICQ) values are equal to the ratio of the number of positive PDM values to the total number of pixel values. From this ratio, 0.5 is subtracted to yield ICQ values distributed between −0.5 and +0.5 where random co-localization gives an ICQ of ∼ 0, segregated or asynchronous co-localization gives −0.5 < ICQ ≤ 0, and synchronous co-localization yields 0 < ICQ ≤ + 0.5.

### cAMP assay

A375 cells (1 × 10^5^ cells/ml, 100 μl) were seeded in 96-well plates and incubated overnight at 37 °C. On the next day, culture medium was replaced by serum free medium. As indicated in the figure legends, A375 cells were treated with CuB (0.1 μM and 1 μM), Forskolin (10 μM, positive control) and pretreated with H89 (20 μM) or MDL12330A (10 μM) for 1 h followed by CuB treatment, respectively. cAMP was measured using a Cyclic AMP XP™ Assay Kit (Cell Signaling Technology) according the manufacturer's directions.

### Extraction of soluble actin

Soluble actin (referred as G-actin hereafter) was extracted as described previously [12]. Briefly, cells were treated with CuB and pretreated with SP600125 (20 μM) or NAC (10 mM) for 1 h followed by CuB (0.1 μM) treatment, respectively. Then, the cells were washed twice with cold PBS (4°C) and G-actin was extracted with soluble actin extraction solution (containing 0.2% Triton X-100) from the cells. The residues (F-actin) were lysed using 2×loading buffer for sodium dodecyl sulfate-polyacrylamide gel electrophoresis (SDS-PAGE).

### Cell proliferation assay

Cell proliferation was measured by MTS assay using the CellTiter 96 Aqueous ONE Solution kit (Promega, Madison, WI). Briefly, cells were seeded into 96-well plates at a density of 4×10^4^/ml and 100 μl/well for 24 h. On the next day, culture medium was replaced. A375 cells were treated with CuB (0.01 μM, 0.1 μM and 1 μM) alone or pretreated with SP600125 (20 μM) for 1 h followed by CuB treatment for 48 h then measured using MTS assays. MTS reagent (20 μl) was added to each well and incubated at 37°C for 1 h–4 h. The absorbance at 490 nm was measured using a microplate reader (Model 680; Bio-Rad, Richmond, CA). Three independent experiments were performed, each in triplicates.

### Western blot analysis

Samples were prepared as described above or by lysing PBS-washed cells with RIPA buffer (Beyotime, Haimen, China). The proteins were separated by SDS-PAGE followed by electro-transfer to polyvinylidene difluoride membrane (Hybond-P; GE Healthcare Life Sciences, Piscataway, NJ). The membrane was probed using antibodies against phospho-VASP (1∶1000), VASP (1∶1000), phospho-cofilin (1∶1000), cofilin (1∶1000), β-tubulin (1∶2000) (Cell Signaling Technology), Gα13 (1∶2000; Abgent), RhoA (1∶2000; Abgent) and pan-actin (1∶2000; Santa Cruz Biotechnology, Santa Cruz, CA), followed by a horseradish peroxidase (HRP)-conjugated second antibody (1∶10000) (Jackson ImmunoResearch, West Grove, PA). Bands were revealed with enhanced chemiluminescence kit (BeyoECL Plus; Beyotime) and recorded on X-ray films (Kodak; Xiamen, Fujian, China). The densitometry of each band was quantified by FluorChem 8000 (AlphaInnotech, San Leandro, CA).

### RNA interference assay

Small interfering RNA (siRNA) duplexes targeting VASP (5′-GGACCUACAGAGGGUGAAAdTdT-3′) were designed and synthesized by RiboBio (Guangzhou, China). The siRNA duplexes (#6267) targeting cofilin was obtained from Cell Signaling Technology. The siRNA of Gα13 (#RI12255) and RhoA (#RI14534) were obtained from Abgent (Suzhou, China). Transfection was performed using N-TER Nanoparticle siRNA Transfection System (Sigma-Aldrich) according to the manufacturer's protocol. In brief, 1×10^4^ A375 cells were plated in 35-mm dishes and cultured overnight. VASP (20 nM), Cofilin (40 nM), Gα13 (40 nM), RhoA (40 nM) and negative control siRNA (20 nM) were transfected into cells, respectively. After 72 h incubation at 37°C, the silencing efficiency was determined by Western blot using specific antibodies. After knockdown for 72 h, the effect of CuB on VASP phosphorylation and actin cytoskeleton were measured by western blot and immunofluorescence microscopy.

### Statistical analysis

All experiments were performed in triplicate, with one representative experiment shown. Data were expressed as mean ± SD. Statistical analysis was performed using GraphPad Prism 4.0 (GraphPad Software Inc., San Diego, CA). One-way ANOVA, followed by Dunnett's multiple comparison tests (versus control), was used to analyze the statistical significance among multiple groups. *P* values < 0.05 were considered statistically significant.

## Results

### CuB-induced VASP phosphorylation and clustering correlate with actin aggregation

**Figure 1:**
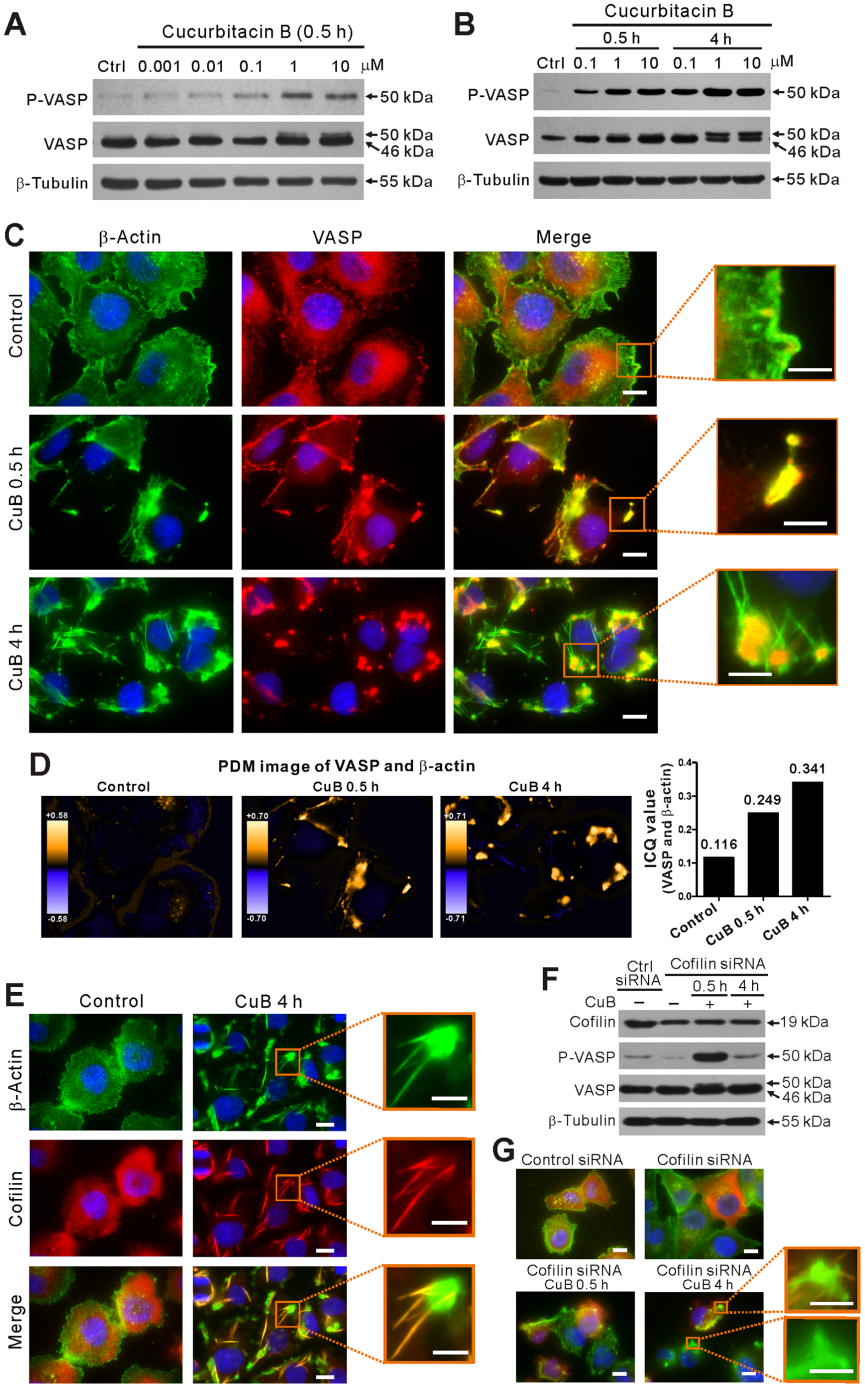
Cucurbitacin B (CuB) induced VASP phosphorylation and clustering in A375 cells. (A,B) Western blot analysis showing the dose- (A) and time- (B) dependent effect of CuB on VASP phosphorylation. (C) Immunofluorescence microscopy analysis showing the co-localization (yellow) of VASP (red) and actin (green) with nuclei (blue) counter staining after 0.1 μM CuB treatment. (D) Co-localization analysis of VASP and actin of C. Both PDM images and ICQ values (right panel) are shown. (E) CuB induced cofilin-actin rod formation. Cells were immunostained with anti-β-actin (green) and anti-cofilin (red) antibodies with nuclei (blue) counter staining. (F) Influence of cofilin knockdown on CuB-induced VASP activation. (G) Influence of cofilin knockdown on actin distribution. Magnified images of the boxed areas (merged images) are presented in C, E and G. Scale bars: 10 μm (5 μm in magnified images).

Whereas CuB is well known for its actin cytoskeleton-disrupting activity in various cancer cells [9], [12], [14]–[16], the underlying mechanism is incompletely understood. In view of the critical role of VASP in linking signaling pathways to the actin cytoskeleton remodeling, we tested whether this protein is involved in the process of CuB-induced actin aggregation. The function of VASP has been shown to be regulated by phosphorylation at three distinct residues (serine 157 (Ser157), serine 239 (Ser239), and threonine 278 (Thr278) residues) [30]. In this study, we showed that VASP was phosphorylated at Ser157 by CuB treatment in a time- and dose-dependent manner in both A375 (Figure 1, A and B) and B16F10 (Figure S1A in File S1) melanoma cells. The phosphorylation of VASP at Ser157, but not Ser239 or Thr278, led to an electrophoretic mobility shift from 46 to 50 kDa in SDS-PAGE [23], [30]. These results suggest that VASP may be involved in CuB-induced actin aggregation.

Next we used immunofluorescence microscopy to explore the localization of VASP in CuB-treated cells. Whereas VASP was diffusely distributed and only slightly co-localized with the actin at the leading edge as well as punctuate actin in control cells, it was locally clustered and co-localized with actin aggregates (hereafter referred to as VASP-actin clusters) upon CuB treatment for 0.5 h (Figure 1, C and D; Figure S1B in File S1). Four hours later, most of the VASP formed large aggregates which were still co-localized with the amorphous actin aggregates, but unexpectedly, not with the rod-like actin structures (Figure 1C). Concomitant with the aggravation of CuB-induced actin aggregation (Figure 1C), the levels of phosphorylated VASP were markedly increased over time (Figure 1B). Notably, we found that those rod-like actin structures were highly co-localized with cofilin rods (Figure 1E), in agreement with previous observation that these rods were actually cofilin-actin rods [17], [22]. In cofilin siRNA-transfected cells, CuB-induced VASP phosphorylation was even enhanced at 0.5 h, and then returned back to similar level to control later (4 h) (Figure 1F). Meanwhile, the cofilin-actin rod formation was substantially suppressed when cofilin was knocked down (Figure 1G). Interestingly, although the cofilin-actin rods did not co-localize with the VASP-actin clusters, it seemed that most of them were protruded from these clusters (Figure 1C, 4 h and Figure 1D). These results suggest that the actin aggregation, as well as the remodeling of amorphous actin clusters into cofilin-actin rods by CuB treatment, is dynamically regulated by VASP activation.

### VASP is required for the reorganization of CuB-induced actin aggregates into cofilin-actin rods

**Figure 2:**
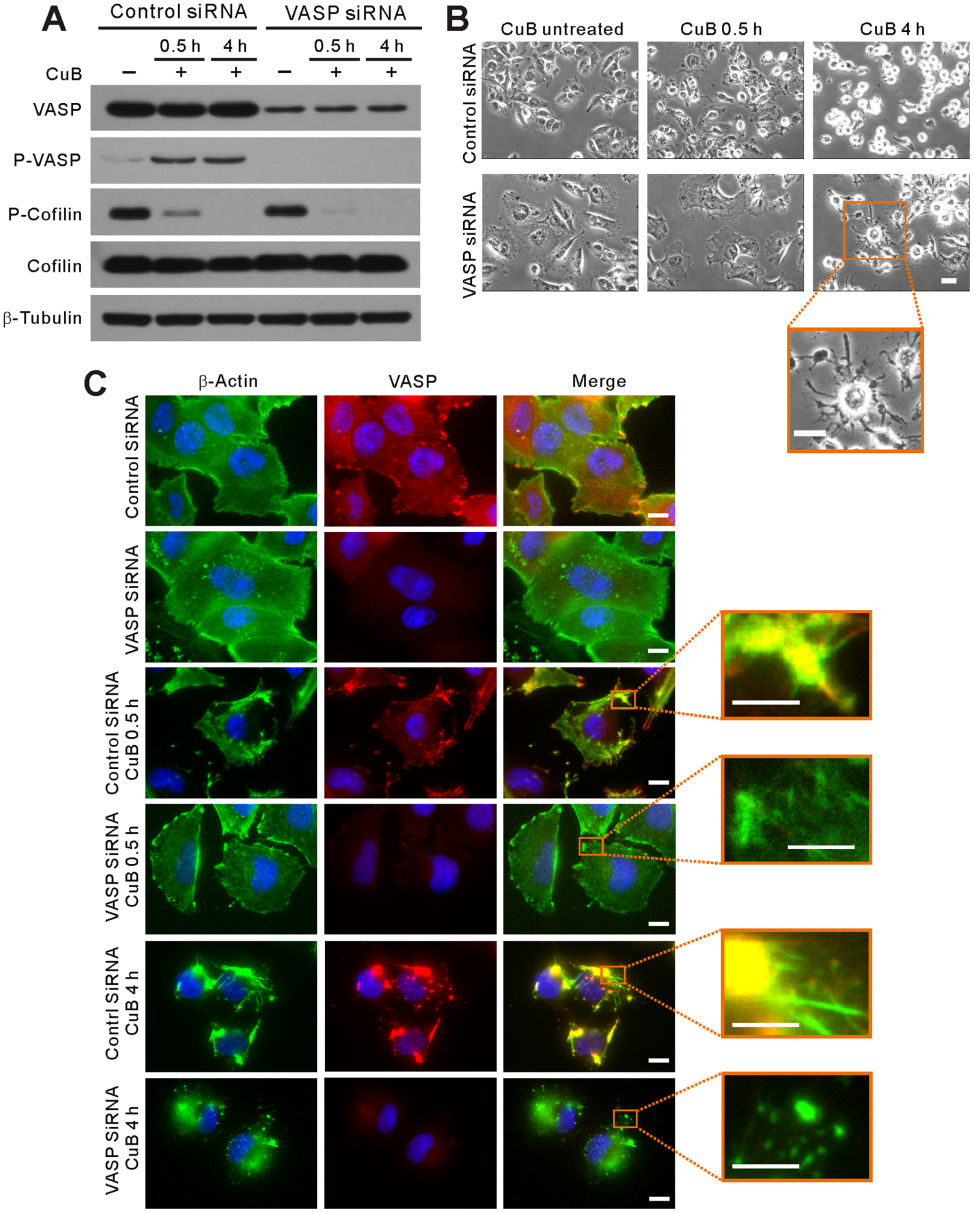
VASP knockdown mitigated CuB (0.1 μM)-induced actin aggregation, cofilin-actin rod formation and cell deformation. (A) Knockdown of VASP did not affect CuB-induced cofilin dephosphorylation as revealed by western blot analysis. (B) Effect of VASP knockdown on CuB-induced cell deformation as observed using phase-contrast microscopy. Scale bars: 20 μm. (C) Immunofluorescence microscopy analysis of the influence of VASP (red) knockdown on CuB-induced actin (green) aggregation. Scale bars: 10 μm (5 μm in magnified images).

As CuB treatment caused cell deformation and actin aggregation followed by remodeling actin aggregates into cofilin-actin rods, we further explored the role of VASP in these processes by siRNA knockdown of its expression in A375 cells. Western blotting showed that VASP levels were reduced by nearly 80% at 72 h post transfection (Figure 2A). Knockdown of VASP slightly enlarged the cell size as observed by phase contrast microscopy (Figure 2B). When cultured with CuB for 0.5 h, the cell deformation was significantly suppressed in VASP-knockdown cells compared with that in controls. Notably, 4-h CuB treatment generated dendritic-like cells in VASP siRNA-transfected group in contrast to oval or rounded cells in control siRNA-transfected sample (Figure 2B, 4 h). Consistent with these results, immunofluorescence microscopy revealed that knockdown of VASP, hence reducing its activation, markedly diminished CuB-induced actin aggregation upon 0.5-h exposure to CuB treatment and fully abolished actin rod formation after 4-h CuB treatment, although actin aggregation still took place (Figure 2C). Unexpectedly, cofilin activation was unchanged upon VASP knockdown (Figure 2A), indicating that the activation of cofilin is insufficient, though indispensable, to induce cofilin-actin rods by CuB treatment. These results also suggested that VASP had participated in the formation of actin aggregates upon CuB treatment and was necessary for reorganizing those amorphous actin clusters into highly-ordered cofilin-actin rods.

### CuB-induced VASP phosphorylation is mediated by cAMP-independent PKA activation

**Figure 3:**
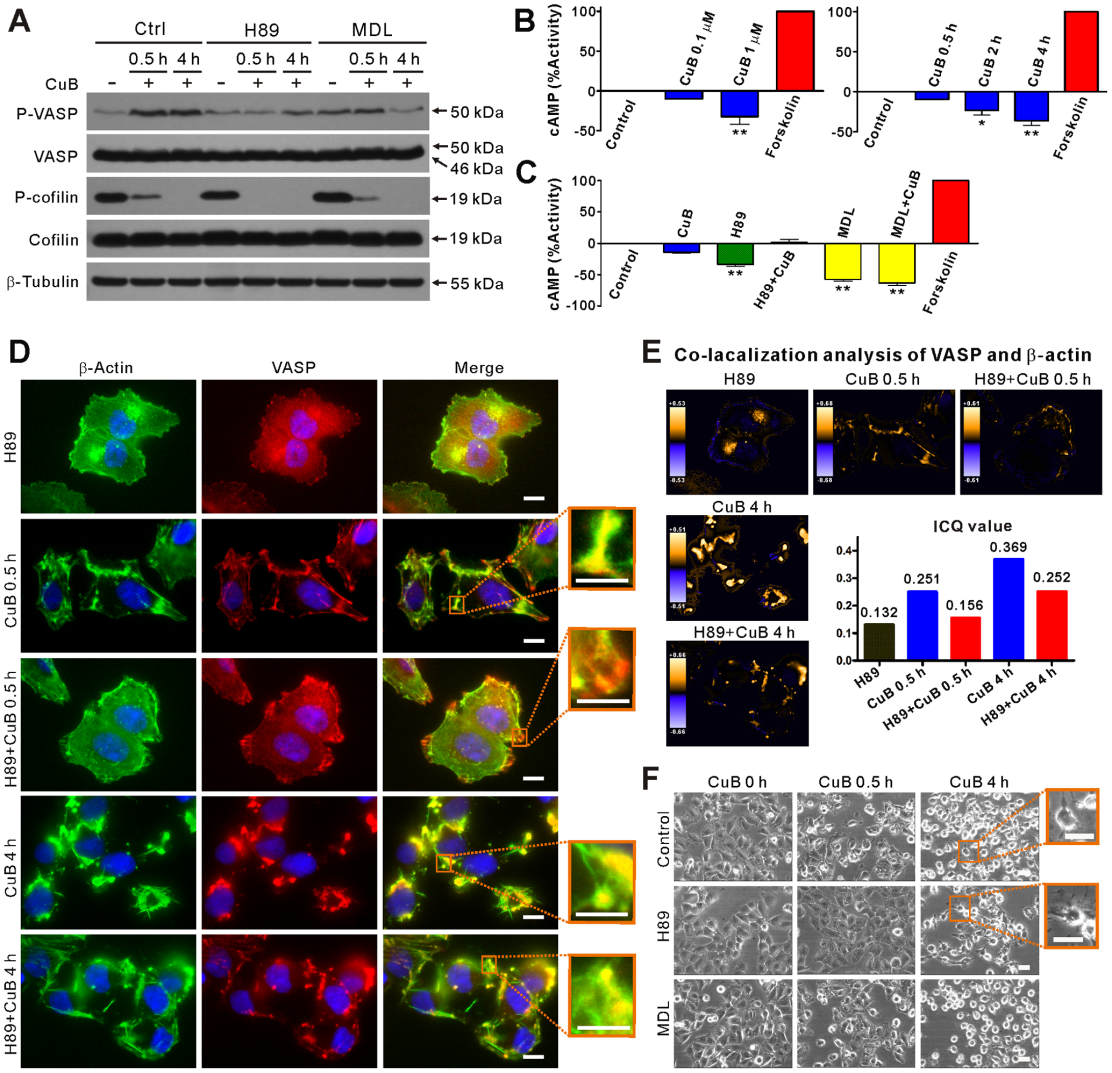
H89, but not MDL12330A (MDL), pretreatment suppressed CuB-induced VASP activation, actin aggregation and cell deformation. (A) Western blotting showing the effects of H89 and MDL pretreatment on CuB-induced VASP activation and cofilin dephosphorylation. (B,C) CuB decreased the levels of cAMP in a time- and dose-dependent manner (B). H89 and MDL pretreatment affected the levels of cAMP in CuB-treated cells (C). The cAMP level of forskolin (10 μM)-treated cells was set as 100% activity (B,C). (D) Immunofluorescence analysis of the influence of H89 pretreatment on the distribution of VASP (red) and actin (green) in CuB-treated cells. Scale bars: 10 μm (5 μm in magnified images). (E) Co-localization analysis of VASP and actin of D. (F) Effect of H89 pretreatment and MDL pretreatment on CuB-induced cell deformation as observed using phase-contrast microscopy. Magnified images of the boxed areas are presented on the right. Scale bars: 20 μm.

It has been reported that Ser157 residue of VASP is preferentially phosphorylated by the cAMP-dependent protein kinase A (PKA) [31]. To determine whether cAMP/PKA signaling is involved in CuB-induced VASP phosphorylation, A375 cells were pretreated with H89, a selective inhibitor of PKA, or MDL12330A, an adenylyl cyclase inhibitor that blocks cAMP production, prior to CuB treatment. The phosphorylation of VASP (Ser157) was subsequently analyzed. The results showed that CuB-induced VASP phosphorylation was profoundly suppressed by H89 pretreatment (Figure 3A). MDL12330A pretreatment also suppressed CuB-induced VASP phosphorylation, but was less effective compared to H89. In B16F10 cells, H89 also inhibited CuB-induced VASP phosphorylation, whereas MDL12330A did not (Figure S1C in File S1). These observations prompted us to consider whether CuB induces the production of cAMP. Surprisingly, cAMP level was decreased by CuB treatment in a time- and dose-depended manner (Figure 3B). As expected, both H89 and MDL12330A pretreatment caused a decrease of cAMP in control cells; MDL12330A pretreatment further decreased the production of CuB-induced cAMP (Figure 3C), but VASP phosphorylation remained at a high level (Figure 3A, 0.5 h). These results indicate that the kinase activity of PKA, being independent of cAMP and adenylyl cyclase activity, is required for CuB-induced VASP phosphorylation at Ser157 residue.

To further investigate the functional consequence of PKA inhibition, we analyzed the effect of H89 on CuB-induced aggregation of VASP and actin using immunofluorescence microscopy. Blocking the kinase activity of PKA with H89 and thereby suppressing the activation of VASP (Figure 3A) effectively inhibited actin and VASP aggregation upon 0.5-h CuB exposure (Figure 3D, Figure S1D in File S1). The formation of CuB-induced cofilin-actin rod was also suppressed by H89 pretreatment (Figure 3D). Although at 4-h CuB incubation it was slightly higher than control, the ICQ value at 0.5-h CuB incubation in the presence of H89 was comparable to the control treated with H89 alone (Figure 3E), indicating that H89 reduced CuB-induced co-localization between VASP and actin. Furthermore, similar to the action of VASP-knockdown, H89 pretreatment suppressed CuB-induced cell deformation at 0.5 h, and resulted in more dendritic-like cells at 4 h of CuB exposure. By contrast, MDL12330A pretreatment did not affect CuB-induced cell deformation (Figure 3F). Together, although H89 was unable to fully block actin aggregation, these results demonstrate that CuB-induced actin aggregation is mainly mediated by cAMP-independent PKA/VASP activation.

### Gα13 and RhoA are upstream components of CuB-induced PKA/VASP signaling

**Figure 4:**
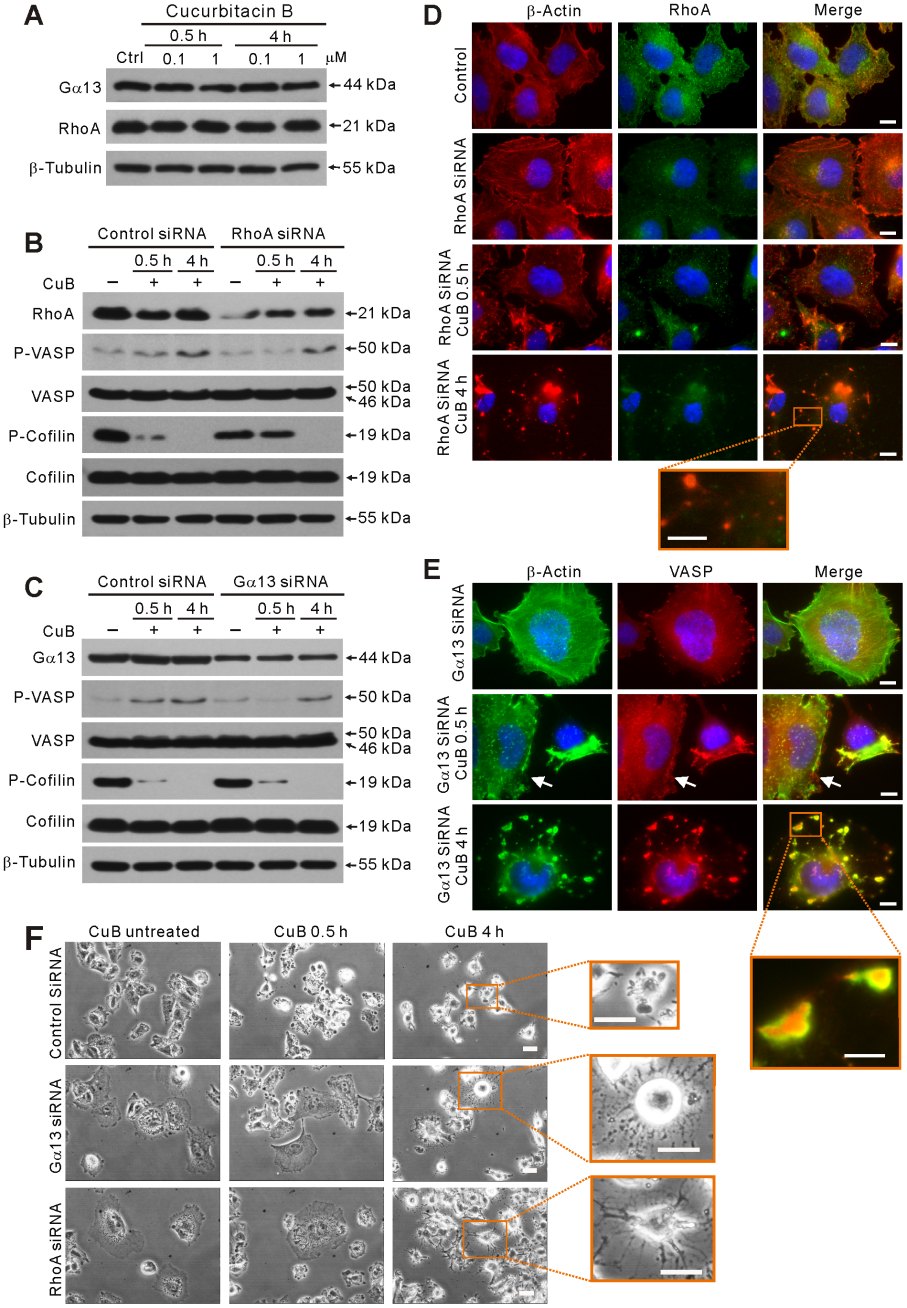
Gα13 and RhoA were involved in CuB-induced VASP activation and actin cytoskeleton disruption. (A) The expression of Gα13 and RhoA was not affected after CuB treatment as evidenced by western blotting. (B,C) Western blot analysis showing the effect of RhoA (B) or Gα13 (C) knockdown on CuB-induced VASP activation and cofilin dephosphorylation (B,C) and JNK activation (C). (D,E)Immunofluorescence microscopy analysis showing the distribution of RhoA (green) and actin (red) in of RhoA-knockdown cells (D) and the distribution of VASP (red) and actin (green) in Gα13-knockdown cells (E). Scale bars: 10 μm (5 μm in magnified images). The white arrowheads indicate the cell in which Gα13 was knocked down (E). (F) Effect of Gα13 and RhoA knockdown on CuB-induced cell deformation observed using phase-contrast microscopy. Scale bars: 20 μm.

Next, we explored whether Gα13/RhoA signaling, which has been reported to function in modulating VASP activity [32], is involved in regulating CuB-induced and PKA-mediated phosphorylation of VASP at Ser157 residue. Initially, western blot analysis showed that the protein levels of Gα13 and RhoA were not affected by CuB treatment (Figure 4A). However, when Gα13 and RhoA genes in A375 cells were knocked down by siRNA, CuB-induced VASP activation was diminished as compared with control siRNA-treated cells (Figure 4, B and C). Moreover, knockdown of RhoA, but not Gα13, temporarily rescued cofilin dephosphorylation after exposure to CuB for 0.5 h (Figure 4B), suggesting that RhoA signaling participated in CuB-induced cofilin activation. Interestingly, knockdown of either RhoA or Gα13 markedly enlarged the cell size (Figure 4, D-F), and profoundly suppressed the actin aggregation (Figure 4D) and cell deformation (Figure 4F) induced by CuB treatment for 0.5 h. The cofilin-actin rods which should be induced by 4-h CuB treatment were also abrogated by either RhoA or Gα13 knockdown (Figure 4, D and E). Similar to the effect of VASP-knockdown, RhoA- or Gα13-knockdown resulted in scattered distribution of actin aggregates (Figure 4D) and dendritic-like cells (Figure 4F) upon CuB treatment for 4 h. Together, these results indicate that CuB-induced VASP activation is mediated by the Gα13/RhoA/PKA signaling pathway.

## Discussion

Emerging evidence indicates that cucurbitacin B (CuB), like other cucurbitacins, induces a rapid damage to the actin cytoskeleton in tumor cells. Although it has been intensively explored by researchers for many years, the underlying mechanism is still not well understood [9], [12], [15], [19]. We reported here that Gα13/RhoA/PKA signaling-mediated VASP phosphorylation plays a critical role in CuB-induced actin aggregation and cofilin-actin rod formation. Such actin-damaging effects of CuB, as well as PKA-mediated phosphorylation at Ser157 residue of VASP in response to CuB treatment, are either significantly suppressed or fully blocked upon knockdown of Gα13 or RhoA gene expression. These findings are in agreement with the previous report that Gα13-regulated RhoA and PKA activity controls cytoskeletal rearrangement via a cAMP-independent mechanism [32].

The mammalian Ena/VASP proteins, including VASP, have important roles in linking signaling pathways to remodeling of the actin cytoskeleton [24], [33]. VASP translocation to the cell periphery depends on Ser157 phosphorylation, while regulation of F-actin assembly by VASP does not require the phosphorylation of its Ser157 residue [30]. High local concentrations of VASP, or high ratio of VASP to actin, may intensify the regulatory activity of VASP in actin reorganization [34]. Concomitant with CuB-induced VASP phosphorylation at Ser157, we observed that VASP was firstly accumulated and aggregated in the cell periphery where actin aggregates initiated upon CuB treatment. Subsequently, the aggregated VASP was closely co-localized with actin aggregates. Afterwards, cofilin-actin rods protruded from the VASP-actin clusters. Such distinct redistribution patterns of VASP upon CuB treatment suggest that VASP may be involved in remodeling the actin aggregates into highly-ordered cofilin-actin rods. This notion is supported by the observations that VASP activation was positively correlated with the severity of actin cytoskeleton disruption and G-actin pool depletion and that VASP knockdown markedly suppressed CuB-induced actin aggregation and completely blocked the cofilin-actin rod formation. Consistent with previous studies indicating that VASP promotes G-actin incorporation at growing filament ends [24] while phosphorylation of VASP at Ser157 reduces its ability to bind to G-actin [25], [33], our results indicate that VASP plays a crucial role in CuB-induced actin cytoskeleton reorganization.

Considering the importance of VASP activation in disruption of the actin cytoskeleton organization by CuB treatment, we have also attempted to identify the upstream components controlling the phosphorylation of VASP. Consistent with previous observations [31], CuB-induced VASP phosphorylation at Ser157 was mediated by PKA since it could be inhibited by the selective PKA inhibitor H89. PKA is well known to be activated by cAMP, which is generated by adenylyl cyclase [35]. However, CuB treatment did not increased but instead decreased the levels of cAMP in cells. Furthermore, CuB-induced VASP activation was not very sensitive to adenylyl cyclase inhibitor MDL12330A, which markedly decreased cAMP production. Therefore, CuB-induced phosphorylation of VASP at Ser157 was mediated by cAMP-independent PKA activation.

**Figure 5:**
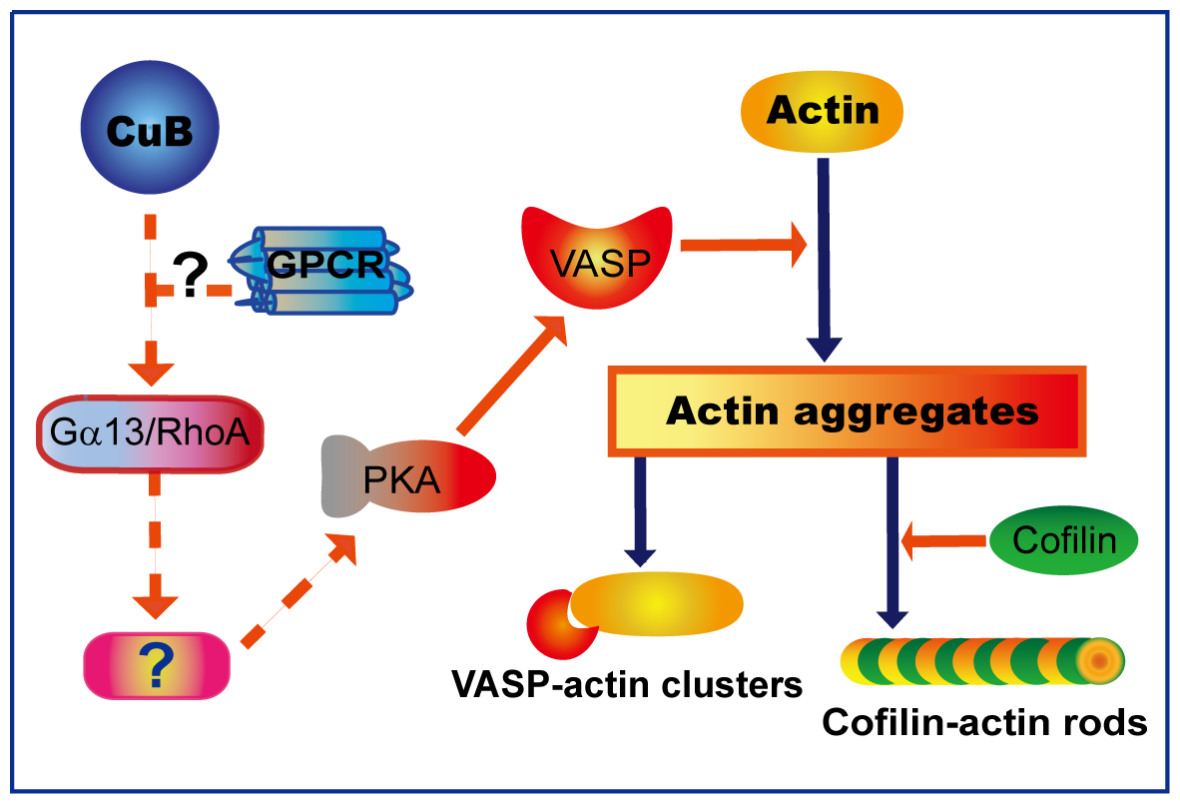
A proposed mechanism depicts CuB-induced actin aggregation and VASP activation via the Gα13/RhoA/PKA pathway. Dashed arrows and question marks indicate unidentifed/unconfirmed signaling components.

It has been reported that, in addition to being activated by cAMP, PKA can also be activated by the Gα13/RhoA pathway, leading to the phosphorylation of VASP [32]. Gα13 is a member of the large family of G-proteins that are linked with G-protein coupled receptors (GPCRs) to transmit extracellular stimuli to intracellular signaling cascades [36], [37]. Among the G-proteins, Gα13 mediates the signaling of Rho (RAS homology) family members that regulate reorganization of the actin cytoskeleton [36]–[38]. Interestingly, we found that CuB-induced VASP phosphorylation at Ser157, actin aggregation and cell deformation were all inhibited either by RhoA knockdown or by Gα13 knockdown. These results established that CuB-induced VASP activation was mediated by PKA activation via the Gα13/RhoA signaling pathway. Further supporting our observation, actin cytoskeleton disruption induced by cucurbitacin IIa and cucurbitacin I is associated with increased activity of RhoA [11], [39]. Moreover, due to the critical role of Rho proteins in regulating actin cytoskeleton, bacterial toxins targeting these proteins lead to actin aggregation similar to the action of CuB [40]. However, it is still unclear how cAMP-independent PKA was activated by CuB-induced Gα13/RhoA signaling. It has been reported that Gα13-induced VASP phosphorylation may lead to activation of RhoA and MEKK1, phosphorylation and degradation of IκB, release of PKA catalytic subunit from the complex with IκB and NF-κB, and subsequent phosphorylation of VASP [32]. Whether similar mechanism is involved in CuB-induced PKA activation needs further investigation. Collectively, we provided evidence that the Gα13/RhoA/PKA axis plays a critical role in mediating CuB-induced VASP activation, resulting in actin aggregation and cofilin-actin rod formation (Figure 5).

In summary, we demonstrated that VASP activation mediated by the Gα13/RhoA/PKA pathway plays a crucial role in CuB-induced actin aggregation and cofilin-actin rod formation. However, it is still unknown how Gα13 is activated by CuB. Notably, the chemical structure of the skeleton of cucurbitacin type tetracyclic triterpenoids is similar to that of other sterols [37], [41], such as cholesterol (Figure S2 in File S1). Cucurbitacins, including CuB, can compete for ecdysteroid receptors in insects [18]. Although ecdysteroid receptors are not present in mammalian cells, a number of mammalian GPCRs are reportedly modulated by cholesterol, the most abundant component of eukaryotic membranes [42], [43]. Thus, it is probable that CuB as well as other cucurbitacins may act on certain GPCRs linked with Gα13 (Figure 5). Further works are warranted to clarify the exact GPCR(s) targeted by cucurbitacins.

## Supporting Information

Figure S1 **CuB-induced VASP phosphorylation (activation) was mediated by PKA in CuB-treated B16F10 cells.** (**A**) Western blotting showing that VASP was rapidly phosphorylated in a dose- and time-dependent manner in CuB-treated cells. (**B, D**) Immunofluorescence microscopy analysis of VASP (red) and actin (green) in CuB (1 μM) -treated cells in the absence (B) or presence of PKA inhibitor H89 (D). Colocalization analysis of VASP and actin was performed and both PDM images and ICQ values (lower panel) are shown. Scale bars: 10 μm (5 μm in magnified images). (**C**) Effect of H89 or MDL12330A (MDL) pretreatment on CuB-induced VASP activation. CuB, 0.5 h.
(DOC)

Figure S2 **Chemical structure of CuB and cholesterol.** (**A**) The basic skeleton of cucurbitane. (**B**) Chemical structure of CuB. (**C**) Chemical structure of cholesterol.
(DOC)
