# Supplementary material for: VASP Activation via the Gα13/RhoA/PKA Pathway Mediates Cucurbitacin-B-Induced Actin Aggregation and Cofilin-Actin Rod Formation
Source: PLoS One. 2014 Apr 1;9(4):e93547. doi: 10.1371/journal.pone.0093547 (PMC3972149; doi:10.1371/journal.pone.0093547)
Supplement: Figure S1 — CuB-induced VASP phosphorylation (activation) was mediated by PKA in CuB-treated B16F10 cells. (A) Western blotting showing that VASP was rapidly phosphorylated in a dose- and time-dependent manner in CuB-treated cells. (B, D) Immunofluorescence microscopy analysis of VASP (red) and actin (green) in CuB (1 μM) -treated cells in the absence (B) or presence of PKA inhibitor H89 (D). Colocalization analysis of VASP and actin was performed and both PDM images and ICQ values (lower panel) are shown. Scale bars: 10 μm (5 μm in magnified images). (C) Effect of H89 or MDL12330A (MDL) pretreatment on CuB-induced VASP activation. CuB, 0.5 h. (DOC) [file pone.0093547.s001.doc]

**Supporting Information**


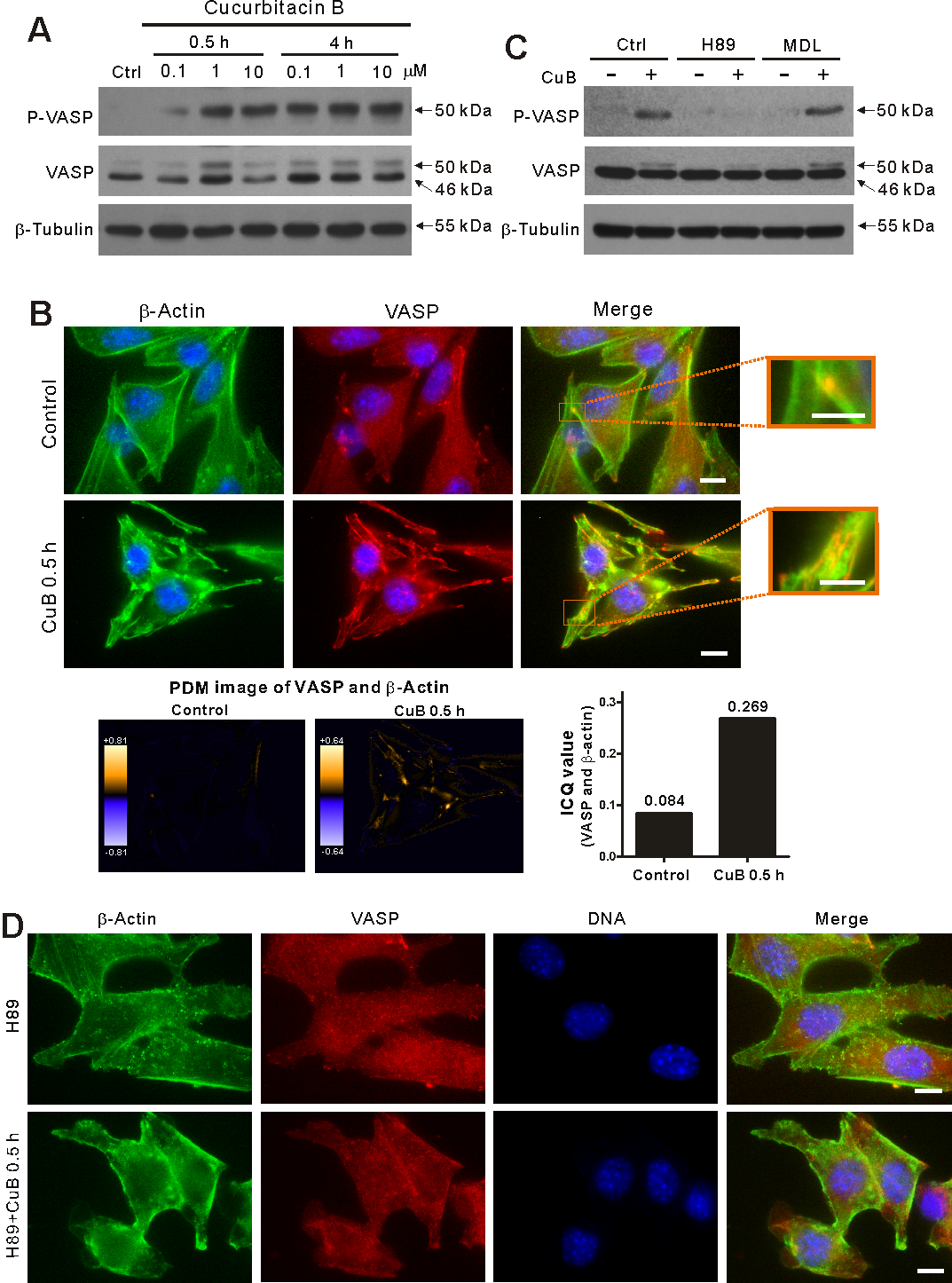


**Figure S1. CuB-induced VASP phosphorylation (activation) was mediated by PKA in CuB-treated B16F10 cells.** (**A**) Western blotting showing that VASP was rapidly phosphorylated in a dose- and time-dependent manner in CuB-treated cells. (**B, D**) Immunofluorescence microscopy analysis of VASP (red) and actin (green) in CuB (1 μM) -treated cells in the absence (B) or presence of PKA inhibitor H89 (D). Colocalization analysis of VASP and actin was performed and both PDM images and ICQ values (lower panel) are shown. Scale bars: 10 μm (5 μm in magnified images). (**C**) Effect of H89 or MDL12330A (MDL) pretreatment on CuB-induced VASP activation. CuB, 0.5
