# Supplementary material for: VASP Activation via the Gα13/RhoA/PKA Pathway Mediates Cucurbitacin-B-Induced Actin Aggregation and Cofilin-Actin Rod Formation
Source: PLoS One. 2014 Apr 1;9(4):e93547. doi: 10.1371/journal.pone.0093547 (PMC3972149; doi:10.1371/journal.pone.0093547)
Supplement: Figure S2 — Chemical structure of CuB and cholesterol. (A) The basic skeleton of cucurbitane. (B) Chemical structure of CuB. (C) Chemical structure of cholesterol. (DOC) [file pone.0093547.s002.doc]

**Supporting Information**


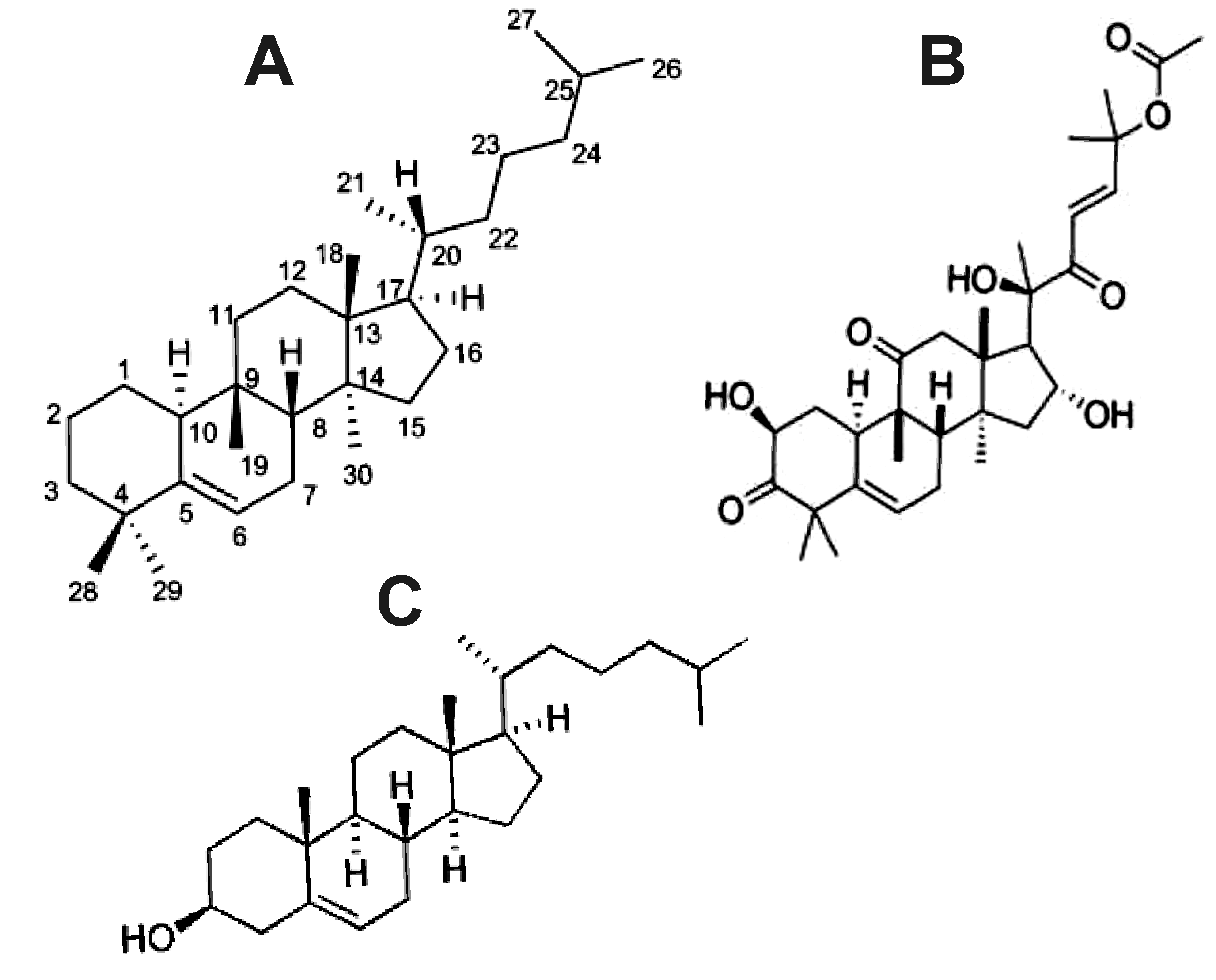


**Figure S2** **Chemical structure of CuB and cholesterol.** (**A**) The basic skeleton of cucurbitane. (**B**) Chemical structure of CuB. (**C)** Chemical structure of cholesterol.
